# Exploring pUS27: Insights into Its Role in HCMV Pathogenesis and Potential for Antiviral Strategies

**DOI:** 10.3390/pathogens14100993

**Published:** 2025-10-01

**Authors:** Gage M. Connors, Juliet V. Spencer

**Affiliations:** Division of Biology, Texas Woman’s University, Denton, TX 76204, USA; gconnors1@twu.edu

**Keywords:** HCMV, cellular GPCR, vGPCR

## Abstract

Human cytomegalovirus (HCMV) is a complex pathogen that encodes a diverse array of proteins essential for its survival and replication within host organisms. Among these proteins, a noteworthy group comprises four chemokine-like G protein-coupled receptors (cellular GPCRs), which play pivotal roles in the virus’s evasion of the host immune response and the establishment of persistent infections. Of particular interest is pUS28, recognized as one of the most extensively studied viral GPCRs (vGPCRs). This receptor has attracted significant attention for its potential as a target for innovative antiviral therapies aimed at addressing HCMV-related diseases. In contrast, pUS27 has not been as thoroughly characterized, presenting a potentially promising avenue for antiviral intervention. The relative scarcity of research surrounding pUS27 underscores an exciting opportunity for further exploration, as a deeper understanding of its functions and mechanisms may reveal novel strategies for combating HCMV infections. This review seeks to synthesize recent advancements in our understanding of pUS27, elucidating its biological roles, interactions, and potential implications for therapeutic development. We will also highlight critical gaps in the existing literature that warrant further investigation, underscoring the need for a more comprehensive understanding of this understudied receptor. By delving into the complexities of pUS27, we aim to inspire future research initiatives that could lead to the development of novel antiviral treatments, thereby enhancing our overall understanding of HCMV pathogenesis. Importance: The study of vGPCRs is essential for understanding how viruses like HCMV manipulate host cell signaling and evade immune responses. While pUS28 has been extensively studied due to its broad chemokine binding and signaling activity, its lesser-known homolog, pUS27, warrants closer attention. Likely arising from a gene duplication event, pUS27 shares approximately 31% sequence identity with pUS28 and is conserved across HCMV strains, suggesting an important functional role. By focusing on pUS27, we may uncover shared mechanisms that allow therapies to effectively target both pUS28 and pUS27, potentially leading to more potent antiviral treatments. The implications of studying pUS27 are profound, as it could play a pivotal role in improving our approaches to combating HCMV and enhancing our overall understanding of immune evasion strategies.

## 1. Introduction

HCMV is a widespread pathogen, with prevalence rates nearing 100% in regions such as Africa and Asia, and approximately 80% in Europe and North America [1]. A member of the β-herpesvirus family, HCMV is species-specific and can infect a wide range of cells, including human fibroblast cells, epithelial cells, endothelial cells, smooth muscle cells, and myelocytes [2]. Following primary infection, HCMV can establish latency, typically in hematopoietic progenitor cells (HPCs) and cells of the myeloid lineage. During latency, productive viral gene expression is silenced through epigenetic modifications, modulation of cellular signaling, and control of viral and cellular microRNAs. While healthy individuals often remain asymptomatic during this latency, HCMV poses significant risks to immunocompromised patients, particularly those undergoing organ transplantation, potentially resulting in severe complications such as end-organ disease, as the virus can reactivate. Furthermore, HCMV is recognized as the most prevalent congenital infection worldwide, affecting one in every 200 newborns, with about 20% of these infants at risk of developing long-term complications, including blindness, hearing loss, and microcephaly [3]. The virus’s capacity to establish lifelong latency and reactivate during immune-compromised states, coupled with the vulnerabilities of immunocompromised and immune-naïve populations, underscores its clinical significance.

HCMV’s ability to successfully infect and establish lifelong latency, as well as promote viral fitness in its host, is due to a number of factors, one being the more than 200 viral proteins it encodes. HCMV encodes four vGPCRs with homology to a subclass of mammalian cellular GPCRs known as chemokine receptors. Cellular GPCRs are vital to a multitude of physiological processes by signaling in cells to regulate most cellular outcomes, including chemotaxis, where they act as primary sensors for chemoattractants and chemical signals that guide cell movement [4]. They also play critical roles in immune function, particularly in leukocyte trafficking [5]. Given their essential contributions to various biological processes, cellular GPCRs have emerged as a major focus in pharmacology, accounting for approximately 30% of all FDA-approved therapeutics [6]. Since their discovery in the 1990s, much research has gone into vGPCRs and their role in HCMV infection. Currently, HCMV is known to express four vGPCRs: pUS27, pUS28, pUL33, and pUL78. Among these, pUS28 is the most thoroughly characterized, emerging as a promising target for antiviral strategies due to its multifaceted roles during infection, including the support of viral latency and reactivation, modulation of immune responses, and involvement in cardiovascular disease [7,8,9,10]. pUL33 and pUL78 also play a role in the reactivation of HCMV from latency, with pUL33 signaling inducing cellular cyclic AMP response element binding protein (CREB1) phosphorylation, a transcription factor that promotes reactivation [11], while Gαi coupling via the DRL motif of pUL78 is essential for efficient reactivation from latent infection [12]. Both pUL33 and pUL78 also contribute to HCMV pathogenesis by facilitating immune evasion, while pUL33 aids in dissemination in fibroblasts, but only in the Merlin strain of HCMV and not in TB40E or AD169 [13,14,15], with pUL33 exhibiting oncomodulatory properties [16]. pUS27 is required for the extracellular spread of the virus [17], and also enhances cell proliferation [18]. However, this vGPCR appears to contribute far more to HCMV fitness than previously recognized, as discussed below.

## 2. Structure of pUS27 vs. pUS28

**Figure 1:**
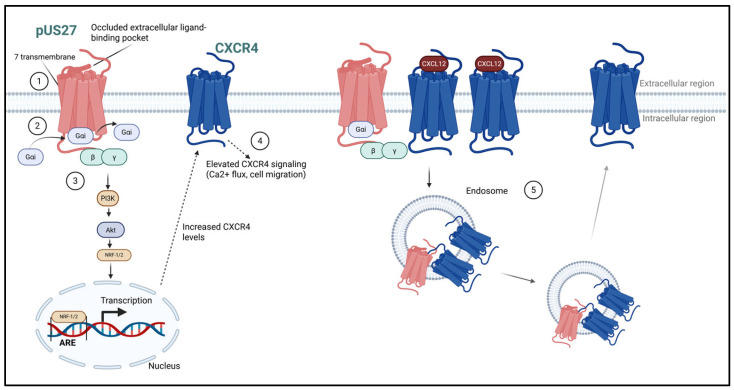
Visual Summary of Recent pUS27 Discoveries. Part 1, cartoon illustration of pUS27 structure depicting occluded binding pocket. Part 2, Gαi weak binding affinity for pUS27. Part 3 depicts the signaling pathway for ARE-regulated CXCR4 expression levels. Part 4 shows the increased calcium flux regulated by pUS27s influence on CXCR4 signaling. Part 5, pUS27 increases CXCR4 internalization and slows cell surface recovery.

Recent investigations into pUS27 have led to intriguing insights regarding its classification and functional capabilities. Traditionally regarded as an orphan receptor, pUS27 lacks any known binding ligands. Structurally, pUS27 features a characteristic seven-transmembrane domain architecture, typical of G-protein-coupled receptors. Its transmembrane helices are arranged in a manner that creates an occluded extracellular ligand-binding pocket (Figure 1), which may explain its inability to engage with ligands [19]. This anatomical feature raises compelling questions about the evolutionary trajectory of pUS27, indicating that it may have adapted to signal through G-proteins in a constitutive manner, independent of ligand interactions [20]. This is an interesting feature of pUS27 when compared to pUS28, which has the ability to signal in both a ligand-dependent and a constitutive manner [21,22,23,24,25,26]. Along with pUS28’s ability to signal through a number of chemokines, including fractalkine, RANTES, MCP-3, and MIP-1α [21,22,23,24,25,26], which play significant roles in immune signaling and responses, pUS28 can also constitutively activate key transcription factors, including NF-κB and cyclic AMP response element-binding protein (CREB) [27]. This activation occurs in a ligand-independent manner, which means that pUS28 can influence downstream gene expression even in the absence of its natural ligands. Interestingly, pUS28 is also constitutively endocytosed, which has become a potential anti-viral target for HCMV using fusion toxin protein (FTP) technology [28]. Another intriguing fact of pUS27 and pUS28 is that they are produced as a polycistronic transcript during lytic infection, but it remains uncertain how, of the two, only the pUS28 transcript is expressed during latency [29]. Indeed, this is another area for exploration. Table 1 summarizes the known signaling and functionality of both pUS27 and pUS28. The implications of this functionality are significant, particularly in the context of immune evasion mechanisms employed by the virus.

## 3. pUS27 Binds Gαi Proteins and Signals Through Gβγ

Similar to pUS28, pUS27 engages Gαi proteins [19,30], a characteristic feature of cellular GPCRs that facilitates signaling through these G-proteins to elicit various cellular responses. Interestingly, pUS27’s interaction with inactive Gαi proteins is marked by a weak binding affinity (Figure 1) [19], suggesting it may function as a decoy, sequestering Gαi away from its typical interactions with other cellular GPCRs on the cell surface. In this same study, they also suggest a Gαi decoy effect for pUS28 [19]. This decoy effect could potentially diminish the availability of Gαi for signaling pathways generally activated by chemokine receptors. To substantiate this hypothesis, further experimental studies are warranted to assess the impact of pUS27 on Gαi protein levels in infected cells.

While the role pUS27 plays as a Gαi decoy is intriguing, there is another aspect of its G-protein interaction that unveils a novel signaling pathway activated by this vGPCR. Recent research has demonstrated that pUS27 signals constitutively through Gβγ and phosphoinositide 3-kinases (PI3K), leading to the activation and nuclear translocation of Nuclear Respiratory Factor 1 (NRF-1) [30]. This translocation enhances the expression of genes regulated by the Antioxidant Response Element (ARE), including C-X-C chemokine receptor type 4 (CXCR4) (Figure 1) [30], which could aid in HCMV immune evasion by stimulating stress response genes. Moreover, pUS27 elevates CD47 expression during viral infection [30]. CD47 functions in immune evasion by transmitting inhibitory signals to macrophages, thereby preventing phagocytosis. An upregulation of CD47 expression could consequently reduce the likelihood of macrophage-mediated clearance of infected cells.

pUS27’s ability to activate the NRF-1/ARE pathway leads to the upregulation of various stress response genes. This can help the virus survive under stressful conditions, such as oxidative stress from the host’s immune response, thereby enhancing viral replication. More studies are needed to determine whether other ARE-regulated genes are affected by pUS27, such as Heme Oxygenase-1 (HO-1), which could help the virus mitigate oxidative damage during infection, supporting viral replication and persistence. As research continues, it is anticipated that additional signaling pathways activated by pUS27 will be uncovered, further elucidating its multifaceted role in viral pathogenesis and immune modulation.

A critical consideration arises from the apparent lack of a deactivation mechanism for the signaling pathways mediated by pUS27. Given that it would be counterproductive for the virus to express a signaling protein without a means to regulate its activity, this raises an essential question: how does pUS27 modulate its signaling processes? Understanding the regulatory mechanisms at play is vital for elucidating the broader implications of pUS27 in viral pathogenesis and its potential role in immune evasion as well as the mechanisms pUS27 employs to regulate its signaling. Given the exploitation of constitutively endocytosed pUS28 using FTP technology as an anti-viral approach to combat HCMV, [28] it would be beneficial to understand the mechanisms pUS27 uses in its constitutive endocytosis, which could also be a target by this methodology.

## 4. pUS27 Regulates CXCR4 Signaling, Endocytosis, and Recycling

CXCR4, a prominent member of the chemokine receptor subfamily of cellular GPCRs, is characterized by its seven transmembrane domains, much like other cellular GPCRs. The primary ligand for CXCR4 is CXCL12, also referred to as SDF-1. This receptor plays a critical role in various physiological processes, including the recruitment of leukocytes, the development of the nervous system during embryonic development, and serving as a co-receptor for the human immunodeficiency virus (HIV) [36].

Studies have highlighted the influence of viral proteins on CXCR4 signaling dynamics [37,38,39,40]. Notably, the viral protein pUS27 is a modulator of CXCL12-mediated calcium mobilization [41], suggesting a complex interplay between viral components and host cell signaling pathways. Furthermore, pUS27 is implicated in the regulation of CXCR4’s endocytosis and recycling processes [42]. Studies using mouse fibroblast cells co-expressing pUS27 and CXCR4 revealed that pUS27 enhances the internalization of CXCR4 in response to CXCL12 stimulation and delays the recovery of CXCR4 to the cell surface (Figure 1) [42]. The co-localization of pUS27 and CXCR4 at the plasma membrane and within the cell indicates they may share similar endocytic pathways [42].

The capacity of pUS27 to modulate CXCR4 recycling dynamics raises intriguing questions about its potential role in promoting the migration of infected cells toward CXCL12-expressing tissues, such as bone marrow, where HCMV establishes latency. This could enhance the mobilization of infected cells to the bone marrow, thereby facilitating the establishment of a more extensive reservoir of infected cells within the hematopoietic compartment. This strategic accumulation of infected cells could not only aid in the persistence of the virus within the host but also contribute to its evasion of immune surveillance mechanisms. However, further research, particularly studies on HCMV infection, is necessary to elucidate the interactions between pUS27 and other HCMV-encoded proteins and their collective impact on CXCR4 dynamics.

## 5. Discussion

HCMV represents a complex viral entity characterized by its exceptional capacity to modulate the host’s immune response while establishing a state of lifelong latency. This intricate nature of HCMV poses significant challenges in the pursuit of effective vaccine development. To enhance vaccine strategies, it is imperative to attain a comprehensive understanding of the mechanisms by which HCMV manipulates host cellular signaling pathways and physiological responses during the course of infection. Current research endeavors concerning HCMV encompass not only the formulation of preventive vaccines but also the exploration of antiviral treatments for individuals already infected with the virus. A particularly noteworthy aspect of HCMV pathogenesis is the involvement of its vGPCRs, which have emerged as promising targets for antiviral interventions.

Among these, the vGPCR pUS28 has attracted considerable attention due to its critical role in the virus’s overall fitness and disease processes. In contrast, pUS27, while less extensively studied than pUS28, also plays significant roles in HCMV infection and warrants investigation as a potential target for antiviral therapies. However, one complicating factor in considering pUS27 as an antiviral target, in comparison to pUS28, is its apparent orphan status concerning ligands. The ability to utilize ligand binding capabilities is a key aspect of targeting both cellular GPCRs for drug therapies and vGPCRs as antiviral agents. Nonetheless, it is noteworthy that methodologies currently employed, particularly in the context of pUS28, could be adapted for pUS27 and its constitutive mechanisms. For instance, small molecules such as benzamide, arylamines, and piperazinyldibenzothiepins have demonstrated efficacy as pUS28 antagonists in in vitro signaling assays [43]. Additionally, certain compounds, including methiothepin and octoclothepin, have been shown to partially inhibit the constitutive activity of pUS28 [44].

It may be beneficial for future studies to investigate the potential of developing a drug capable of inhibiting the constitutive activities of both pUS27 and pUS28, thereby enhancing the likelihood of effectively mitigating HCMV pathogenesis. Another promising avenue of research involves the utilization of viral G protein-coupled receptor-targeting nanobodies. In 2018, researchers successfully developed a US28-targeting nanobody (US28-Nb) by immunizing a llama and an alpaca with DNA encoding the vGPCR, resulting in a sub-micromolar affinity for displacing US28 ligands. By genetically linking two US28-Nbs with a flexible linker, they created a bivalent US28-Nb that exhibited a 100-fold increase in binding affinity and potency, effectively inhibiting US28’s constitutive activity by up to 50% [45]. Studies exploring these types of drugs that target the constitutive activities of vGPCRs could extend beyond pUS28 and pUS27 to include pUL78 and UL33, which is also classified as an orphan receptor.

## 6. Conclusions

In conclusion, this review has highlighted recent advancements in our understanding of the pUS27 protein and its multifaceted role in the pathogenesis of HCMV. Notably, pUS27 has been shown to regulate ARE-associated genes, a mechanism that may facilitate immune evasion, as well as influence the internalization and recovery of CXCR4—processes that are intimately linked to viral dissemination and latency. Furthermore, the interaction of pUS27 with inactive Gαi proteins suggests a potential decoy mechanism that could contribute to immune evasion strategies employed by the virus.

While significant progress has been made, numerous questions persist regarding the precise mechanisms through which pUS27 promotes immune evasion and viral persistence. Continued investigation into these pathways is imperative, as it may lead to the development of innovative therapeutic strategies aimed at disrupting HCMV’s ability to circumvent host immune responses and maintain latency. As our comprehension of the roles played by HCMV-encoded vGPCRs expands, the prospects for devising targeted antiviral therapies become increasingly promising. This evolving body of knowledge underscores the importance of ongoing research in elucidating the complex interactions between HCMV and the host immune system.

## Figures and Tables

**Table 1:** Comparison of Signaling, Functions, and Ligands between pUS27 and pUS28.

Feature	pUS27	pUS28
Signaling Pathways	Limited G protein engagement; interacts with Gαi but does not activate signaling [19] Signals through Gβγ and PI3K to activate ARE genes [30]	Activates multiple G proteins including Gαq, G 12/13, and Gαi [31] Strongly recruits β-arrestin for alternative signaling routes [32]
Constitutive Activity	Exhibits high basal activity despite impaired Gαi coupling [19] Constitutive activation of NRF-1 and CXCR4. [30]	Displays robust constitutive activity [31] Promiscuous chemokine-binding profile enhances viral manipulation of host signaling pathways [33]
Chemokine Selectivity	No known chemokine ligands identified [31]	Broad-spectrum chemokine receptor, capable of binding multiple chemokines from different classes (CC, CXC, XC, CX3C) [31]
Functional Role	Modulates host immune signaling and promotes extracellular viral dissemination [17,30]	Contributes to immune evasion and viral dissemination [34,35] Critical for latency establishment and maintenance by interfering with host signaling [31] US28’s full list of functional roles is summarized nicely in [31]
Structural Characteristics	Occluded extracellular binding pocket restricts interaction with ligands [19]	Flexible extracellular domain enables binding of diverse host chemokines [19]

## Data Availability

Not applicable.
